# Screening and brief intervention for patients with tobacco and at-risk alcohol use in a dental setting

**DOI:** 10.1186/1940-0640-7-S1-A19

**Published:** 2012-10-09

**Authors:** Bonnie McRee, Thomas Babor, Frances Del Boca, Janice Vendetti, Cheryl Oncken, Howard Bailit, Joseph Burleson

**Affiliations:** 1 Department of Community Medicine, University of Connecticut School of Medicine, Farmington, CT, USA

## 

Despite the relevance of screening and brief intervention (SBI) to the prevention of dental pathology, particularly with tobacco and at-risk alcohol use, there has been little attention to the determination of its effectiveness in dental settings. Further, most SBI research efforts have focused on the treatment of single risk factors despite the fact that use of psychoactive substances tends to co-occur. There is also debate about the optimal timing of interventions for multiple risk behaviors, i.e., whether to intervene simultaneously or sequentially. This study was designed to test the efficacy of SBI practices aimed at dental patients who were both smokers and at-risk drinkers. Participants (N = 288) were randomized into four experimental conditions to test the efficacy of comparative interventions for tobacco and at-risk alcohol use when delivered separately and in combined forms, and to compare the effects of sequential versus simultaneous interventions. The results indicated that individuals in each of three active brief intervention (BI) groups (alcohol BI, tobacco BI, and combined alcohol and tobacco BI) significantly reduced self-reported drinks per week and cigarettes per day compared with those in the wait-list control group. There was no advantage to the combined versus single-substance focused interventions as individuals changed both behaviors regardless of the treatment intervention received. No significant differences in self-reported drinks per week or cigarettes per day were found between those receiving simultaneous versus sequential interventions. These findings have implications for the design of BI aimed at multiple substance use and imply that no matter where a provider begins with respect to behavior-change focus, she or he may affect change in patients across multiple substance use behaviors.

